# Capacitation‐Induced Zinc Ion Flux and Sperm Plasma Membrane Remodeling Predict Porcine In Vitro Fertilization Cleavage Success

**DOI:** 10.1002/mrd.70085

**Published:** 2026-01-21

**Authors:** Isabel Rodriguez, Alexandra Keller, Lindsey Jennett, Megan Johnson, Ian Shofner, Mubashrah Mahmood, Bethany Redel, Karl Kerns

**Affiliations:** ^1^ Department of Animal Science Iowa State University Ames Iowa USA; ^2^ Department of Genetics and Genomics Iowa State University Ames Iowa USA; ^3^ USDA‐ARS, Plant Genetics Research Unit Columbia Missouri USA

**Keywords:** image‐based flow cytometry, in vitro fertilization, porcine, sperm capacitation, zinc signatures

## Abstract

Semen evaluation in human and animal reproduction relies on sperm motility and morphology; however, these often fail to predict fertility. The domestic boar (*Sus scrofa*) serves as a biomedical model for male reproduction due to similarities in sperm size, capacitation dynamics, and acrosomal structure to humans in comparison to traditional rodent models. This study evaluated sperm capacitation biomarkers, particularly zinc signatures, to predict cleavage success after in vitro fertilization (IVF). Semen from 20 boars (3 replicates each) was analyzed at 0, 1 and 4 h post‐in vitro capacitation (IVC) using image‐based flow cytometry to assess 4 zinc signatures, plasma membrane integrity, and acrosomal remodeling. Capacitation kinetics were quantified between timepoints. Motility was measured by computer‐assisted semen analysis, and IVF cleavage percentages were determined. Zinc signature 1 at 4 h post‐IVC negatively correlated with cleavage percentage (*r* = −0.366), indicating higher noncapacitated sperm proportions reduce fertilization potential. The delta of zinc signature 3 from 1 to 0 h also negatively correlated (*r* = −0.441), suggesting excessively rapid capacitation impairs fertilization. Models combining capacitation biomarkers, motility, kinetics, and morphology parameters had higher predictive power (*R* ^2^ = 0.469) than motility models alone. Zinc signatures may serve as mechanistic fertility biomarkers in a translational boar model applicable to animal breeding and human‐assisted reproduction.

## Graphical abstract

Capacitation‐associated zinc redistribution and membrane remodeling predict IVF cleavage success. Distinct boar ejaculates were subjected to in‐vitro capacitation (IVC) and evaluated using image‐based flow cytometry and computer‐assisted semen analysis (CASA). Zinc ion redistribution and acrosomal membrane remodeling before and after IVC were associated with higher cleavage percentages, whereas dysregulated capacitation dynamics were associated with poor cleavage outcomes, demonstrating that capacitation kinetics, rather than motility alone, are critical predictors of fertility. Created in BioRender. Kerns (2026) https://BioRender.com/3an4sui.

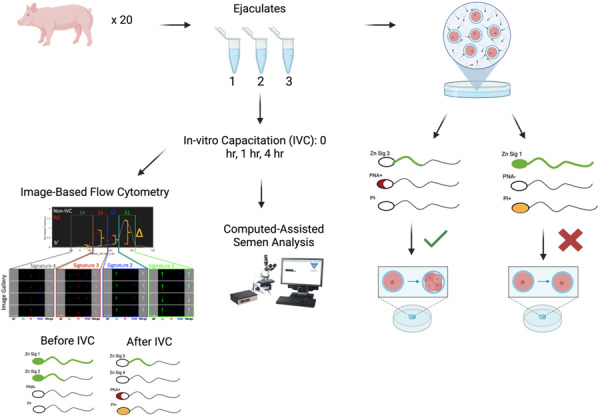

## 1 Introduction

The domestic boar (*Sus scrofa*) is a well‐established biomedical model for human male reproductive physiology, due to its similarities in sperm dimensions, capacitation dynamics, acrosomal structure, and fertilization mechanisms compared to rodent models. Pigs closely mimic human sperm structure and function, making them valuable for studying sperm biology, capacitation, and assisted reproduction under controlled experimental conditions (Mateo‐Otero et al. 2023; Pang et al. 2022; Zigo et al. 2020). Insights from porcine reproduction research can inform both livestock breeding strategies and human clinical approaches to diagnosing and addressing subfertility. In both human and swine populations, semen evaluation commonly relies on sperm motility and morphology; however, these measures alone do not reliably predict fertility potential, with up to 25% of boars meeting conventional semen quality thresholds still exhibiting suboptimal conception rates. Industry data indicate that roughly one‐quarter of boars meeting stringent semen quality thresholds (≥ 80% motility and morphology) still achieve conception rates below 80%, underscoring the limitations of current selection and fertility diagnostic practices (Shike 2020).

Artificial insemination (AI) has emerged as the primary method for breeding in the US swine industry, with over 97% of sows and gilts being impregnated through this assisted reproductive technology (USDA ERS n.d.). The efficiency of AI can be limited by the functional fertility of the sire's spermatozoa (Mellagi et al. 2023). Current industry guidelines for boar ejaculate selection include ≥ 70% gross sperm motility and ≥ 75 morphology (Althouse 2015), though in practice, thresholds are often set as high as 80%.

Sperm motility and morphology have long been used as the male fertility determinant in commercial swine operations. Typically, a computer‐assisted semen analysis (CASA) is employed to assess sperm motion kinematic parameters and as a program to support male sperm quality analysis (Bae et al. 2024). While these parameters are important prerequisites for fertilization, they do not tell the full biological story of a spermatozoon's potential to fertilize an oocyte. Thus, looking into additional parameters to determine boar fertility should be studied.

Following ejaculation, spermatozoa must undergo further maturation in the female reproductive tract to acquire the capacity to fertilize an oocyte. This maturation process, independently described by Austin (1951) and M. C. Chang (1951), is referred to as sperm capacitation. Sperm capacitation involves a series of biochemical and physiological changes, including hyperactivation, alterations to the plasma membrane, and the preparation for acrosome exocytosis (Ickowicz et al. 2012). Differing percentages of sperm in an ejaculate can undergo this capacitation process, including some spermatozoa that capacitate quickly and others that capacitate slowly, resulting in subpopulations of sperm with varying capacitating kinetics. Additionally, the percent in this subpopulation capable of capacitation can vary by sire/donor (human (Ostermeier et al. 2018), mouse (Escoffier et al. 2015)). It is important to note that though sperm capacitation has been studied for years, it is still a molecular process not well understood (Jin and Yang 2016).

Recent research has highlighted the importance of zinc ion flux during sperm capacitation (Kerns, Zigo, Drobnis, et al. 2018). Zinc ions play a significant role in the regulation of various stages of sperm capacitation by acting as a modulator of cellular signaling pathways and enzyme activities (Kerns, Zigo, and Sutovsky 2018; Zigo et al. 2022). Distinct localization patterns of zinc ions (Zn^2+^) have been identified across various species, denoted as zinc signatures, which correspond with early, mid, and late stages of sperm capacitation. In the boar, four zinc signatures have been discovered. Zinc signature 1 is indicative of a noncapacitated sperm cell with Zn^2+^ localized across the head and whole flagellum. Signature 2 reflects early stages of capacitation with Zn^2+^ uniformly found in the head and midpiece of the flagellum. Signature 3 is associated with intermediate to late capacitation stages, with Zn^2+^ localized exclusively in the sperm midpiece. Lastly, Signature 4 reflects a fully capacitated sperm cell distinguished by the absence of Zn^2+^ (Kerns, Zigo, Drobnis, et al. 2018). In addition to zinc efflux, other markers such as propidium iodide (PI) and peanut agglutinin (PNA) provide further insight into the capacitation status of a sperm cell (Kerns, Zigo, Drobnis, et al. 2018). PI is a membrane‐impermeable fluorescent DNA stain (Crowley et al. 2016) that selectively penetrates sperm cells with compromised plasma membranes. PNA is a lectin that specifically binds to exposed sugars on the acrosome of sperm cells undergoing or that have undergone acrosomal remodeling changes (F. P. Cheng et al. 1996). This lectin is typically conjugated with a fluorescent dye for visualization and analysis.

While many targets have been found for diagnosing male fertility, such as plasma proteins (Parvin et al. 2024), genes and gene expression (Cassuto et al. 2024; Junahar et al. 2024), DNA methylation profiles of gene promoters (Miller et al. 2023), small RNAs (Mehta and Singh 2024), reactive oxygen species (Westerman 2020), ubiquitin and thioredoxin SPTRX3/TXNDC8 (Sutovsky et al. 2015), apoptosis, mitochondrial membrane potential, and DNA damage (Ling et al. 2022), correlations between these biomarkers and embryo development is a field yet to be explored in the pig. To the best of our knowledge, capacitation‐related proteomic changes have been studied in pigs in correlation with increased litter sizes (Kwon et al. 2015). In humans, only a few sperm biomarkers have been studied in correlation with embryo development, such as microRNAs (Conflitti et al. 2023) and sperm proteomic changes (Jodar et al. 2020), but no correlation studies with changes in sperm capacitation ability. This gap in knowledge highlights the need for further research into the role of sperm capacitation status biomarkers in predicting fertility. Thus, the aim of this study is to use sperm capacitation status biomarkers as a predictor of porcine in vitro fertilization (IVF) cleavage percentages.

## 2 Materials and Methods

### 2.1 Experimental Design

A total of 20 randomly selected boars, with 3 replicates each from different ejaculates, were used for this experiment. Different ejaculates per boar were used to account for ejaculate variability. The capacitation status of the samples was evaluated using an image‐based flow cytometer (IBFC) at the following timepoints: 0 h (noncapacitated), 1 h post‐in vitro capacitation (IVC), and 4 h post‐IVC. Delta values between timepoints were calculated as follows: 4 h–0 h, 4 h–1 h, and 1 h–0 h. A CASA was used to assess sperm motility, morphology and kinematic parameters before and after IVC. IVF was conducted to explore potential correlations between capacitation status and embryo development. Each ejaculate was treated as an independent biological insemination event for ejaculate‐level fertility prediction, reflecting real‐world applications where fertilization potential is assessed per ejaculate. At the same time, the repeated‐ejaculate structure within boars was modeled using mixed‐effects approaches, with ejaculates nested within boar ID to account for within‐boar dependency. This approach is supported by established evidence that temporally distinct ejaculates from the same boar differ in sperm function, molecular composition, and fertilizing capacity (Popwell and Flowers 2004) and that spermatozoa undergo continued functional and molecular modifications through ejaculation, producing distinct sperm cohorts across collection events (Y. Chang et al. 2016). These biological considerations motivated the dual analytical structure, enabling ejaculate‐level prediction while ensuring statistical rigor through mixed‐effects modeling.

### 2.2 Semen Preparation and Processing

In total, 20 commercial Duroc boars of the same relative age from a private boar stud were utilized in this study. The semen dosages used were not specifically collected for this study; rather, they were in excess of standard production at the facility. The boars were housed individually and collected once a week using the two‐gloved hand technique. Ejaculates with over 80% motility were selected for processing. Immediately after collection, semen was extended a total of 5 times in Preserve Xtreme extender (GENEPRO, Madison, Wisconsin, United States), ensuring the extender was within 2°C of the semen temperature. Samples were delivered to Iowa State University on the same day of processing. Upon arrival, the temperature of the samples was recorded, and they were stored at 17°C until day of use, which was 3 days post‐arrival.

### 2.3 Capacitation Treatments

Three treatments were applied and analyzed: noncapacitated (0 h), in vitro capacitated (1 h), and in vitro capacitated (4 h). For IVC, 500 μL of each sample was transferred into a 1.5 mL microcentrifuge tube, washed of seminal fluids and sustaining media by centrifugation at 500*g* for 4 min. Sperm pellets were resuspended in 1 mL of modified tris‐buffered medium (mTBM) that was equilibrated in a humidified atmosphere of 5% CO_2_ at 38.5°C for 48 h. Microcentrifuge tubes were tightly closed, sealed from ambient air, and incubated in a water bath at 37° C for either 1 or 4 h.

### 2.4 Computer‐Assisted Semen Analysis (CASA)

For noncapacitated samples (0 h), a 0.5 mL aliquot of each sample was placed in a 1.5 mL microcentrifuge tube and warmed for 20 min at 37° C (Thermo Fisher Scientific, Waltham, Massachusetts, United States). Samples were gently pipetted to ensure even cell distribution and reduce agglutination, and 3.0 µL of each aliquot was loaded into a 20 µm disposable chamber from Minitube. For in vitro capacitated samples (1 and 4 h), immediately after the incubation period in mTBM, 3.0 µL of sample was loaded into a 20 µm disposable chamber from Minitube. Sperm concentration, general motility classifications, kinematic parameters, and morphology were assessed using a Minitube AndroVision CASA. In addition, the CASA system reports composite motility metrics. Total composite score and progressive composite score represent morphology‐weighted motility indices calculated as motility percentage multiplied by the percentage of morphologically normal sperm. Total composite score reflects the proportion of motile sperm that are morphologically normal, and progressive composite score reflects the proportion of progressively motile sperm that are morphologically normal. These composite parameters were included for comparative analysis. The Minitube AndroVision CASA system is coupled to a Zeiss AxioScope 5 microscope fitted with a Basler ace ac2440‐75uc camera and a 10×/0.23 A‐Plan objective lens. The microscope condenser aperture diaphragm was set at 1, and the six‐position filter wheel was set at 2. Videos were acquired at 60 frames per second with a 0.5‐s capture duration per field. For each sample, a minimum of 600 sperm cells were analyzed across 4 nonoverlapping fields. Motility classification was performed using AndroVision software decision‐tree thresholds. Sperm were classified as immotile when amplitude of lateral head displacement was < 1.00 µm and curvilinear velocity (VCL) was < 24.0 µm/s, or when head activity coefficient was < 0.03. Motile sperm were further subdivided, with local motility defined as VSL < 24.0 µm/s and VCL < 48.0 µm/s. Progressive motility included sperm exhibiting either Progressive circular motility, defined by a radius > 10.0 µm and < 30.0 µm with rotation > 0.70, or forward motility characterized as slow motility (VCL < 120.0 µm/s) or fast motility when slow motility criteria were not met.

### 2.5 Image‐Based Flow Cytometry

#### 2.5.1 Flow Cytometry Probe Staining and Incubation

Approximately 5 million spermatozoa from each semen sample were washed of seminal fluids and sustaining media by centrifugation at 500*g* for 4 min. Each sperm pellet was resuspended with 100 μL of a probe media that contained the following: Lectin PNA (*Arachis hypogaea/*PNA) conjugated to Alexa Fluor 647 (Thermo Fisher Scientific) and used at a concentration of 1:2000 from the stock concentration (1 mg/mL) for a final concentration of 0.5 μg/mL; Fluo‐Zin3 AM (FZ3; zinc probe) from Invitrogen (F24195) reconstituted with dimethyl sulfoxide (DMSO) to a stock solution for 500 μg/mL and used at a concentration of 1:500 for a final concentration of 1 μg/mL; Hoechst 33342 (H33342) from Invitrogen (H1399) reconstituted with ddH_2_O to a stock solution of 18 mM and used at a concentration of 1:1000 for a final concentration of 10 μg/mL; PI from Invitrogen (P304MP) reconstituted with ddH_2_O to a stock solution of 1 mg/mL and used 1:1000 for a final concentration of 0.1 μg/mL. The fluorescent labels were incubated with the cells for 30 min at room temperature and protected from light before being washed by centrifugation at 500*g* for 4 min and resuspended in 100 μL of phosphate‐buffered saline (PBS). Spermatozoa were incubated for 30 min at 37°C on a heat block (Fisherbrand), and protected from light, to allow cells to metabolize the probes. After incubation, 80 μL of each sample was loaded onto a 96‐well plate for immediate IBFC analysis.

#### 2.5.2 Image‐Based Flow Cytometry Data Acquisition

A Cytek Amnis ImageStream^X^ (ISX) MkII IBFC was used for data acquisition. The ISX was equipped with a 40× microscope objective with an imaging rate of up to 2000 events/s. The sheath fluid used was PBS with a pH of 7.20. The flow core diameter and speed were 6 μm and 66 mm/s, respectively. Raw image data were acquired using INSPIRE software. Multiple channels were collected, including Bright‐field (Channel 1), FZ3 (Channel 2), PI (Channel 4), one side scatter (SSC) image (Channel 6), H33342 (Channel 7), and PNA‐AF647 (Channel 11). The following lasers and power settings were used: 405 nm (to excite H33342): 10 mW; 488 nm (to excite FZ3): 60 mW; 561 nm (to excite PI): 40 mW, 642 nm (to excite PNA‐AF647): 25 mW; and 785 nM SSC laser: 10 mW.

#### 2.5.3 Image‐Based Flow Cytometry Data Analysis

Data were analyzed using IDEAS analysis software version 6.4. The gating approach included single cells per image that were in focus. Further gating boundaries were determined by population segregation and utilizing the image gallery. Sperm cells that were laterally aligned with the camera (as opposed to posteriorly/anteriorly aligned) were excluded as described (Kerns, Zigo, Drobnis, et al. 2018). Examples of the gating structure and images are included in the Supplemental Materials: zinc signature (Figure S3), PI (Figure S4), acrosome status (Figure S5).

#### 2.5.4 In Vitro Fertilization Media

TL‐HEPES, oocyte maturation medium, oocyte manipulation medium, and mTBM were prepared and stored as described (Redel et al. 2019). NCSU‐37 culture medium (Petters and Wells 1993), supplemented with 0.5 mM calcium lactate and 0.5 mM pyruvate, was filtered using a 0.22 μm filter and prepared on the day of culture. NidOil (NidaCon International AB, Gothenburg, Sweden) overlay was used to prevent evaporation of IVF media in the incubator.

#### 2.5.5 In Vitro Fertilization

Fresh sow ovaries were collected from a local harvest facility and transported to the laboratory. Upon arrival, the ovaries were washed with saline solution maintained at 38.5°C. Follicles ranging from 3 to 5 mm in diameter were aspirated using an 18‐gauge needle attached to a vacuum pump system. The pellet formed from the follicular fluid was washed twice with a TL‐HEPES solution at room temperature. Oocytes containing at least two layers of cumulus cells were selected for maturation and placed in TCM‐199‐based maturation medium supplemented with 0.57 mM l‐cysteine, 10 ng/mL of EGF and FSH, and 0.5 μg/mL of LH (Redel et al. 2019). The oocytes were incubated at 38.5°C in 5% CO_2_ for 40–44 h. Post‐maturation, cumulus cells were removed by pipetting with 0.1% (wt/vol) hyaluronidase for 1 min. Oocytes with a visible polar body were selected and placed in groups of 25–35 oocytes/50 μL droplets of mTBM overlaid with 3 mL of NidOil. The final sperm concentration was adjusted to 0.5 × 10^6^ cells/mL and 50 μL of semen was introduced into each mTBM droplet. Metaphase II oocytes and sperm were coincubated for 4–5 h at 38.5°C in 5% CO_2_. Following fertilization, potential zygotes were washed and placed in a 4‐well Nunc dish containing 500 μL NCSU‐37 culture medium supplemented with 0.5 mM calcium lactate and 0.5 mM pyruvate, overlaid with 250 μL of NidOil. The cells were maintained at 38.5°C in 5% O_2_ for 4 days. Cleavage percentages were visually assessed ~84 h post‐insemination. Embryos exhibiting two or more blastomeres were considered cleaved. Although this timepoint cannot distinguish between parthenogenetic and polyspermic embryos, no activation agents were used, and our IVF system yields consistently low polyspermy percentages (5.5% ± 2.2%). Cleavage percentage was expressed as the percentage of cleaved embryos out of all inseminated oocytes within each replicate.

#### 2.5.6 Semen Preparation for IVF

A 500 μL aliquot of each sample was placed in a 1.5 mL microcentrifuge tube and left at room temperature for 20 min. Concurrently, equilibrated mTBM that was sealed from ambient air was removed from the incubator and allowed to reach room temperature 20 min prior to use. A 150 μL aliquot from each sample was added to 1 mL mTBM and centrifuged in a swinging hinge rotor centrifuge at 500*g* for 5 min. The supernatant was removed, and the sample was resuspended in 1 mL mTBM. This wash step was repeated twice. Sperm concentration was measured using CASA, and each sample was diluted with mTBM to achieve a final concentration of 0.5 × 10^6^ cells/mL.

### 2.6 Statistical Analysis

All statistical analyses were performed using R software version 4.4.2 (R Core Team 2024). The Shapiro–Wilk test was performed to evaluate normality for cleavage percentages and all individual sperm parameters. When both variables were normally distributed, Pearson's correlations were calculated; otherwise, Spearman's rank correlations were applied, and statistically significant correlations were identified. Because each IVF replicate utilized a separate ejaculate, inseminations represent independent biological events at the ejaculate level; however, to ensure appropriate treatment of the hierarchical data structure, downstream analyses were performed both ways by (i) treating ejaculates as independent insemination events and (ii) using mixed‐effects models that included boar ID and ejaculate replicate as random effects. This dual approach preserves the biological relevance of ejaculate‐level prediction while appropriately accounting for within‐boar dependence. Based on cleavage percentages, all inseminations (*n* = 60) were ranked and divided into fertility groups with the highest 20% defined as the high fertility group, and the lowest 20% defined as the low fertility group. Independent *t*‐tests were conducted to test differences in sperm parameters between the two fertility groups.

All sperm parameters were assigned to one of four categories (motility, kinematics, morphology, or biomarkers), and principal component analysis (PCA) was conducted within each category. PC1 scores were used as predictors in fixed‐effects linear regression (LM) models to evaluate their ability to explain variation in cleavage percentage. Two complementary modeling approaches were performed. First, global LM and LMER were fitted using PC1 scores derived from all variable timepoints within each category to assess overall cleavage predictability. Second, timepoint‐specific LM and LMER models were generated using PC1 scores calculated separately for 0, 1, 4 h, and delta values previously described (4 h–0 h, 4 h–1 h, and 1 h–0 h). For each timepoint, category‐specific (motility, kinematics, morphology, biomarkers) and combined values (all four categories combined) were evaluated, and predictive power was quantified using model *R* ^2^ values. Variables used for PCA‐based LM and LMER analysis can be found in Table S3.

The PCA‐based LM was selected as the primary predictive method because it does not require prior knowledge of boar identity or replicate history, which more closely aligns with our goal of representing real‐world applications where fertilization success is estimated based on individual ejaculates without prior history. To verify the robustness of these models, the same PC1 predictors were evaluated using LMER, including boar and replicate as random effects.

## 3 Results

**Figure 1:**
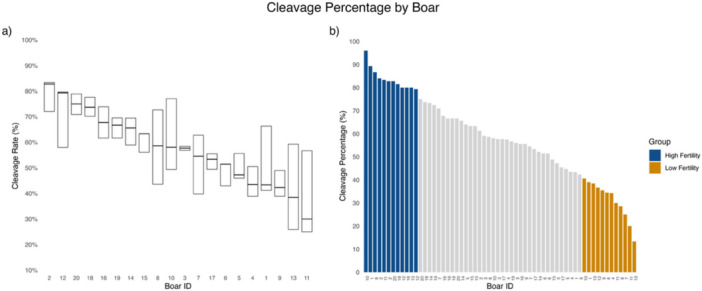
Cleavage percentage distribution across individual boars and fertility group classification. (a) Boxplot showing the distribution of cleavage percentage among the 20 boars, with 3 replicates each. The lower boundary represents the first quartile (Q1), while the upper boundary represents the third quartile (Q3). The middle boundary represents the median. Boars are organized from highest to lowest median cleavage percentage. (b) Ranked bar plot of all 60 inseminations (3 replicates × 20 boars), organized from highest to lowest cleavage percentage. Based on this distribution, inseminations were classified into a high‐fertility group, comprising the top 20% of inseminations, and a low‐fertility group, comprising the bottom 20% of inseminations, as shown in blue and gold, respectively. This classification was used for downstream comparative analyses.

In this study, an assessment of porcine IVF cleavage percentages was evaluated ~84 h postfertilization using neat semen samples from 20 boars, each with 3 replicates from different ejaculates, for a total of 60 inseminations. A broad range of cleavage percentages was observed. Boar 11 displayed the lowest median cleavage percentage, while Boar 2 had the highest median cleavage percentage (Figure 1a). Additionally, cleavage percentage outcomes varied substantially among individual ejaculates from the same boar (Figure 1b).

**Figure 2:**
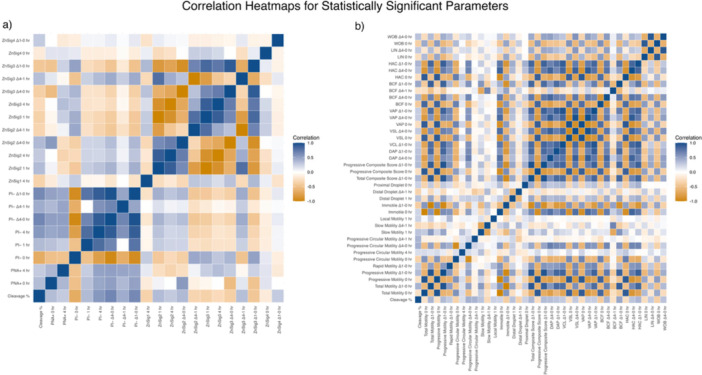
Correlation heatmaps of cleavage percentages and statistically significant parameters. (a) Heatmap representing correlation coefficient (Pearson or Spearman, depending on normality of each parameter pair) between cleavage percentage and biomarkers (Zn signatures, PNA, and PI) measured at 0 h, 1 h post‐IVC, 4 h post‐IVC, and their corresponding delta values. (b) Heatmap representing correlation coefficients between cleavage rates and CASA values, including motility, kinematic, and morphology parameters across 0 h, 1 h post‐IVC, 4 h post‐IVC, and their corresponding delta values. Blue indicates positive correlations with cleavage percentage, while gold indicates negative correlations, with color intensity reflecting the strength of the correlation. Specific correlation test used for each parameter, along with specific correlation coefficients and *p* values, are in Table S1.

Image‐based flow cytometry was used to visualize sperm zinc signatures 1 through 4 throughout the timepoints and delta timepoints previously described. A CASA system was utilized to measure motility, kinematic, and morphology parameters. Correlation coefficients between cleavage percentage, capacitation‐indicative biomarkers (zinc signatures, PI, and PNA status), motility, kinematic, and morphology parameters were evaluated. Out of 206 parameters analyzed, only 66 parameters displayed a significant correlation with cleavage percentage as visualized in Figure 2. A full correlation heatmap, along with corresponding correlation coefficients and *p* values, is provided in Figure S1 and Table S1.

**Figure 3:**
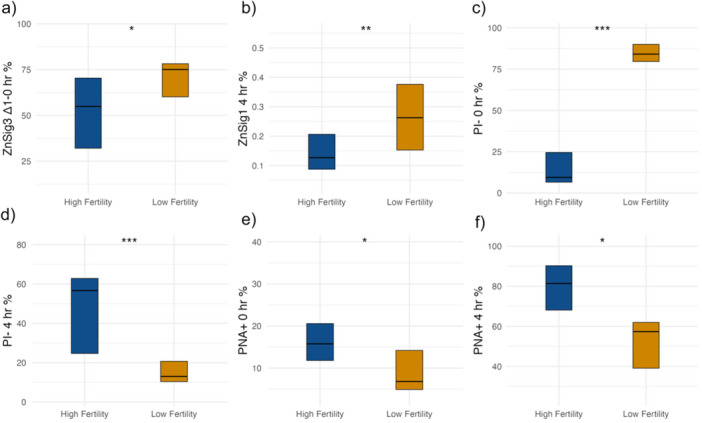
Distribution of sperm capacitation biomarker percentages in high‐ and low‐fertility groups. Fertility groups were defined based on cleavage percentage, with the top 20% of inseminations assigned to the high‐fertility group (blue) and the bottom 20% assigned to the low‐fertility group (gold) (Figure 1b). The biomarkers shown were selected due to their significant correlation with cleavage percentage observed in our prior correlation analysis (Figure 2 and Table S1) and were subsequently evaluated for differences between fertility groups. Independent *t*‐test were performed for each biomarker, and asterisks denote statistical significance (* = *p* < 0.05, ** = *p* < 0.01, *** *= p* < 0.001). The *y*‐axis represents the percentage of the biomarker of interest. (a) Delta value of zinc signature 3 from 1 h post‐IVC to 0 h (ZnSig3_Δ1–0 h). (b) Zinc signature 1 at 4 h post‐IVC (ZnSig1 4 h). (c) PI‐negative cells at 0 h (PI− 0 h). (d) PI‐negative cells at 4 h post‐IVC (PI− 4 h). (e) PNA‐positive cells at 0 h (PNA+ 0 h). (f) PNA‐positive cells at 4 h post‐IVC (PNA+ 4 h).

To further investigate the relationship between sperm parameters and their correlation with cleavage percentage, each IVF insemination was treated as an independent event due to the use of a biologically distinct ejaculate for all inseminations. Ejaculate‐to‐ejaculate variability in motility, capacitation status, and biomarker profiles has been well documented in boar and human reproductive biology, and our data set showed similar variation across ejaculates from the same boar (Figure 1b). From all inseminations (*n* = 60), fertility groups were determined based on cleavage percentage: the top 20% of inseminations with the highest cleavage percentage were classified as the high‐fertility group, while the bottom 20% of inseminations with the lowest cleavage percentage were classified as the low‐fertility group (Figure 1b). Individual *t*‐tests were then performed for each sperm parameter to assess differences between the fertility groups. Several biomarkers identified as significantly correlated with cleavage percentage in our correlation analysis (Figure 2 and Table S1) also differed between fertility groups (Figure 3). For example, zinc signature 1 at 4 h post‐IVC (ZnSig1 4 h) showed a negative correlation with cleavage percentage (*r* = −0.37), and was significantly elevated in the low‐fertility group (*p* = 0.0074; Figure 3b). Similarly, the delta value in zinc signature 3 from 1 h to 0 h (ZnSig3 Δ1–0 h) was negatively correlated with cleavage percentage (*r* = −0.44) and was higher in the low‐fertility group (*p* = 0.0432; Figure 3a).

Acrosomal remodeling also differed between fertility groups and showed a positive correlation with cleavage percentage. PNA‐positive at 0 h (PNA+ 0 h), and PNA‐positive at 4 h post‐IVC (PNA+ 4 h) were each positively correlated with cleavage percentage (*r* = 0.37 and *r* = 0.27, respectively). In both cases, the high‐fertility group exhibited a greater proportion of sperm undergoing acrosomal remodeling than the low‐fertility group (*p* = 0.0289 for PNA+ 0 h (Figure 3e), and *p* = 0.0136 for PNA+ 4 h (Figure 3f)). Plasma membrane integrity also showed fertility‐associated differences. PI‐negative cells at 0 h (PI− 0 h) had a negative correlation with cleavage percentage (*r* = −0.58) and were more abundant in the low‐fertility group (*p* = 8.6 × 10^−7^; Figure 3c). In contrast, PI‐negative 4 h post‐IVC (PI− 4 h) had a positive correlation with cleavage percentage (*r* = 0.48), and exhibited a greater proportion in the high‐fertility group (*p* = 0.0008; Figure 3d). Full *t*‐test results are presented in Table S2.

**Figure 4:**
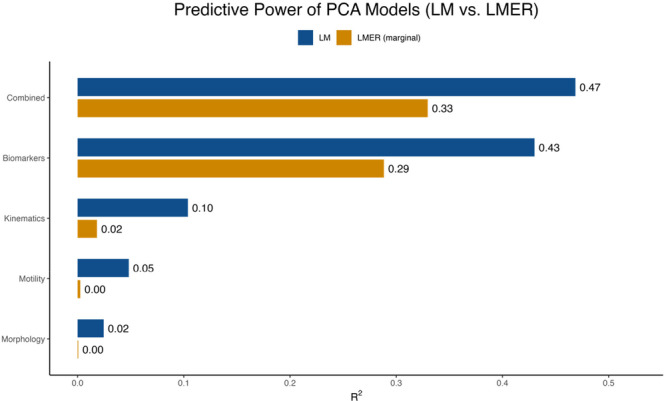
Comparison of predictive power between linear models (LM) and mixed‐effects models (LMER) across sperm parameter categories. Bar plots show the variance explained (*R* ^2^) by LM (blue), and marginal *R* ^2^ from LMER (gold) for motility, kinematics, morphology, biomarkers, and the combined model. LM models consistently explained more variance than LMER models, with the biomarker and combined categories displaying the highest predictive power.

**Figure 5:**
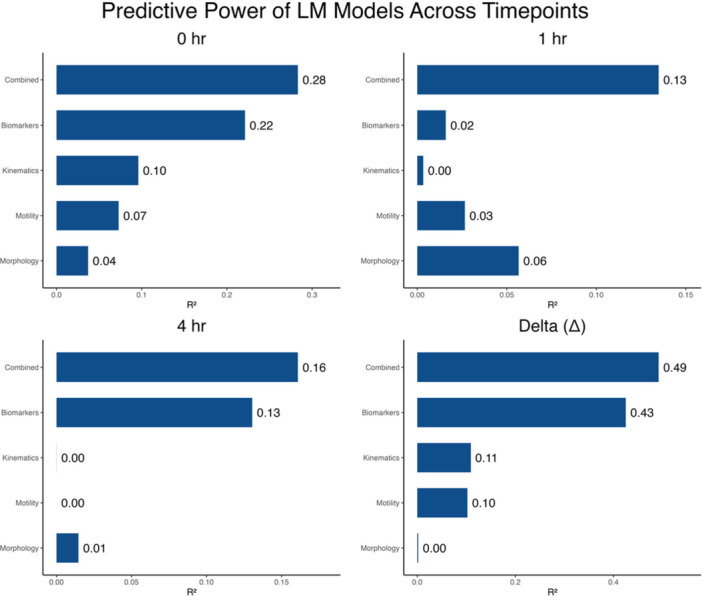
Predictive power of linear models across timepoints. Variance explained (*R* ^2^) by LM models for five parameter categories (motility, kinematics, morphology, biomarkers, and the combined model) evaluated at 0 h, 1 h post‐IVC, 4 h post‐IVC, and for delta values representing dynamic changes between timepoints. Delta models displayed the strongest *R* ^2^ values, indicating that dynamic changes are more predictive than static parameters.

**Figure 6:**
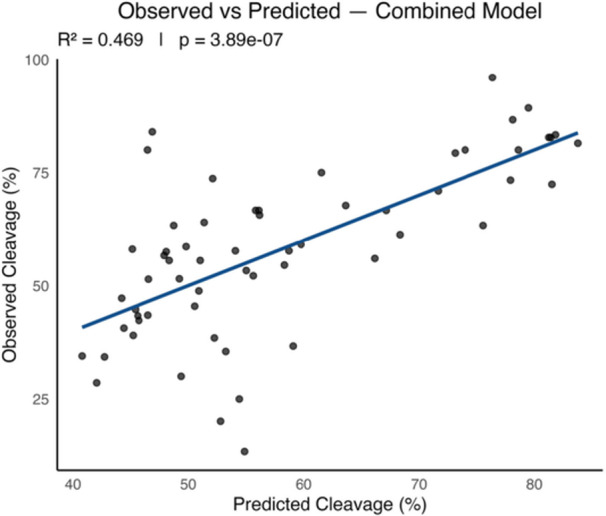
Observed versus predicted cleavage percentages for the combined model. This scatter plot compares observed cleavage percentage with values predicted by the combined linear model using PCA‐derived PC1 scores from all parameter categories. Each dot represents an individual insemination event. The blue regression line indicates the overall trend between predicted and observed values, with the model explaining 46.9% of the variance (*p* = 3.89 × 10^−7^).

To evaluate the predictive power of sperm parameters on cleavage percentage, PCA‐derived PC1 scores from each parameter category (motility, kinematic, morphology, and biomarkers) were incorporated into linear models (LM) and mixed‐effects models (LMER). Across all analyses, LM models consistently outperformed LMER marginal effects, suggesting that ejaculate‐level prediction is stronger when boar identity is not incorporated as a random effect. Among the LM models, the Combined model, which integrates all four parameter categories, achieved the highest overall predictive power (*R* ^2^ = 0.467, *p* = 3.9 × 10^−7^), followed closely by the biomarker‐only model (*R* ^2^ = 0.430, *p* = 1.3 × 10^−8^), whereas motility and morphology models contributed minimally and did not reach statistical significance. Notably, the kinematics model reached statistical significance (*p* = 0.012), but its predictive strength remained low (*R* ^2^ = 0.104), indicating that although detectable, it was insufficient for making meaningful predictions (Figure 4). When models were evaluated at individual timepoints (0 h, 1 h post‐IVC, 4 h post‐IVC, and their corresponding delta values), most timepoint‐specific models performed modestly; however, the Δ (delta) model consistently produced the strongest predictive values (*R* ^2^ = 0.492), indicating that dynamic capacitation changes are more informative than static measurements (Figure 5). Overall, the combined model explained 46.86% of the variance and was highly significant, as visualized in Figure 6. Variables used for analysis are in Table S3, *R* ^2^ values and *p* values for all LM and LMER models are provided in Table S4, and additional LMER visualization across timepoints are presented in Figure S1.

## 4 Discussion

This study aimed to evaluate the predictive power of sperm capacitation status biomarkers on porcine IVF cleavage percentages. It is known that spermatozoa viability can vary between ejaculates (Y. Chang et al. 2016; Petrocelli et al. 2015; Popwell and Flowers 2004) but not necessarily known if fertility varies from ejaculate. Our data reflect that all 20 boars display different levels of fertility from ejaculate to ejaculate, evidenced by the varying cleavage percentages. Figure 1 highlights the variability between ejaculates within individual boars, with some boars showing higher variability than others, emphasizing the unique fertilization potential each ejaculate possesses, even under controlled in vitro conditions.

Our findings show significant correlations between biomarkers of interest (zinc signatures, plasma membrane integrity, and acrosomal remodeling) and IVF cleavage percentages Figure 4. To capture dynamic changes in sperm capacitation over time, delta values were calculated to represent differences in capacitation biomarkers between specific timepoints. Delta from 1 h post‐IVC to 0 h reflects early capacitation events, delta values from 4 h post‐IVC to 1 h post‐IVC represent late capacitation events, and delta values from 4 h post‐IVC to 0 h represent the total capacitation change over the entire 4 h IVC period. These delta values provide insight into the rate and extent of sperm capacitation occurring, which is more informative than looking at singular timepoints alone. By taking these values into consideration, we were able to assess how rapid and/or gradual changes in sperm capacitation affect the cleavage percentage outcome. For example, zinc signature 3 is present in minimal percentages at 0 h; after inducing IVC, the percentage of cells exhibiting zinc signature 3 increases at the 1 and 4 h timepoints. By calculating the change in zinc signature 3 from 4 to 0 h, the focus is placed on the capacitation ability of the sperm cells over time. Similarly, PNA‐positive and PNA‐negative values are inversely related, as are PI‐positive and PI‐negative values; therefore, we report only one value from each pair throughout the manuscript to avoid redundancy. This approach is relevant given the expected physiological changes during sperm capacitation. At 0 h, prior to IVC‐induced changes, sperm cells are expected to have intact plasma membranes (PI‐negative) and no acrosomal remodeling (PNA‐negative). Following IVC, a proportion of sperm cells are expected to exhibit evidence of acrosomal remodeling (PNA‐positive) and signs of compromised plasma membranes (PI‐positive) that are associated with cell death that occurs after late stages of sperm capacitation.

When evaluating the relationship between specific capacitation biomarkers and cleavage percentage, several key findings were observed. Zinc signature 1 at 4 h post‐IVC (ZnSig1 4 h) displayed a negative correlation with cleavage percentage (Figure 2a) and showed a statistical difference between the two fertility groups (Figure 3b). This suggests that a remaining high percentage of noncapacitated sperm cells after inducing capacitation and 4‐h incubation correlates with poorer cleavage rate outcomes. Once capacitation is induced, zinc signature 1 (ZnSig1) percentages typically decrease over time as sperm cells advance through capacitation and adopt different zinc signatures. The persistence of noncapacitated sperm cells after IVC suggests that a high proportion of sperm cells are biologically unable to capacitate successfully, therefore affecting fertility outcomes. Zinc signature 3 reflects mid‐late stages of capacitation. When the delta for 1 h post‐IVC to 0 h (ZnSig3 Δ1–0 h) is evaluated, we are able to assess capacitation‐like changes occurring in a cohort of sperm that have fast capacitation. The negative correlation between ZnSig3 Δ1–0 h and cleavage percentage likely arises due to excessively fast capacitation, resulting in exhaustion of the sperm cell before it is able to fertilize the oocyte. A study on macaque sperm suggests that extreme conditions, such as high alkalinity or energy substrate deprivation in IVC media, may accelerate capacitation but may not be ideal for maintaining sperm vigor and motility over time (Tollner et al. 2009). Additionally, other authors have found increased DNA fragmentation, increased morphological abnormalities, and decreased tyrosine phosphorylation associated with fast capacitation in 1 h versus a slower capacitation over 4 h in humans (Sáez‐Espinosa et al. 2020).

PNA‐positive cells at 0 h (PNA+ 0 h) and PNA‐positive cells at 4 h post‐IVC (PNA+ 4 h) both showed positive correlations with cleavage percentage and had statistical differences between fertility groups. PNA‐positive cells are indicative of acrosomal remodeling, a necessary step during capacitation, but can also reflect poor acrosome health if not previously exposed to capacitating conditions or premature capacitation. The data show that the high fertility group has more sperm cells undergoing acrosomal remodeling than the low fertility group (Figure 3e,f). At 4 h post‐IVC, the majority of the sperm cells should have undergone acrosomal remodeling to prepare for acrosomal exocytosis if they were capacitating successfully. The low fertility group's reduced number of cells undergoing acrosomal remodeling suggests that these cells are not finishing all capacitation‐related steps successfully.

PI‐negative cells at 0 h (PI− 0 h) had a negative correlation with cleavage percentage and showed statistical differences between the two fertility groups. These findings suggest that having a higher initial percentage of sperm cells with intact plasma membranes does not predict higher fertilization success. The high fertility group displays a lower percentage of PI‐negative cells (Figure 3c); in other words, this group had higher counts of PI+ positive cells indicative of compromised plasma membranes or nonviable cells before IVC, which is most likely due to poor epididymal maturation (Rodriguez‐Martinez et al. 2022) or storage. Conversely, PI‐negative cells at 4 h post‐IVC (PI− 4 h) had a positive correlation with cleavage percentage, and we observed that the high fertility group had a higher percentage of cells with intact plasma membranes at the end of IVC (Figure 3d), emphasizing the role of maintaining plasma membrane integrity while undergoing remodeling during capacitation. The low fertility group's reduced percentage of PI‐negative cells at 4 h may indicate that those sperm cells are unable to undergo capacitation optimally, such as too fast, or have other sperm defects.

Overall, when comparing sperm capacitation parameters across fertility groups, a pattern emerges in which balanced and progressive capacitation is favorable for fertilization. Ejaculates with higher cleavage percentage tended to show moderate, well‐timed transitions in capacitation markers, suggesting that sperm progressing through capacitation at an optimal rate retain fertilizing capacity. In contrast, ejaculates with low cleavage percentage often displayed either stalled capacitation (e.g., persistence of zinc signature 1) or excessively rapid transitions (e.g., elevated zinc signature 3 at early timepoints), both of which may compromise functional competence. This implies that both insufficient and overly rapid capacitation are suboptimal, and that a controlled, stepwise capacitation process is a key determinant of successful fertilization. This trend is illustrated in Figure 3a–c reflecting negative correlations, and Figure 3d–f showing positive correlations with cleavage percentage. Given the physiological similarities between boar and human sperm in terms of size, acrosomal structure, and capacitation dynamics, compared to rodent models, the predictive relationships identified here may also have relevance for human assisted reproduction. Notably, sperm zinc signatures are conserved between boar and human, with similar localization patterns observed during capacitation in both species (Kerns, Zigo, and Sutovsky 2018). Incorporating comparable capacitation biomarkers into clinical semen assessment could complement conventional motility and morphology evaluations, potentially improving the detection of subfertility in men who otherwise meet current semen quality thresholds.

When evaluating the predictive power of various parameters on cleavage percentage, different PCA‐based statistical models, such as LM and LMER, were applied to identify the most informative predictors. LMER marginal *R* ^2^ values were considered rather than conditional *R* ^2^ values, as marginal values reflect the variance explained solely by fixed‐effects and are therefore more relevant for real‐world prediction scenarios where boar identity and replicate information are typically unknown. Notably, the LM models consistently explained more variance than the LMER models, supporting their use as the primary predictive framework, while the LMER models served as a model robustness checkpoint.

Our data suggest that the combined model, including biomarker, motility, kinematic, and morphology parameters with all their respective timepoints and delta values, offers a more accurate explanation of the data than the motility, kinematics, and morphology models alone, as evidenced by the *R* ^2^ and *p* values. This suggests that capacitation biomarkers contribute critical information that is not captured by motility, kinematics, or morphology parameters alone. While motility is a necessary prerequisite for fertilization, our findings indicate that it does not account for differences in fertility outcomes after the minimum standard threshold is met. The kinematics model reached statistical significance; however, its predictive strength remained low. The poor predictive performance of the motility and morphology models highlights the limitations of using these parameters alone as a predictor of fertility, a standard currently used regularly by the swine industry. Further supporting these findings, the linear regression analysis performed on the combined model reiterates the statistically significance of this model in fertility prediction.

The findings from this study have practical implications for the swine industry, where AI is the predominant breeding method. Despite meeting standard semen quality thresholds, approximately one in four boars exhibit suboptimal conception rates below 80% (Shike 2020). Incorporating capacitation status biomarkers into routine semen analysis could enhance sire selection and reproductive efficiency, ultimately improving sow herd performance. It is important to note that this study was conducted under in vitro conditions, where the biomarkers associated with successful fertilization may differ from those relevant in vivo. Nonetheless, our results demonstrate that specific sperm biomarkers, particularly those reflecting capacitation dynamics, are reliable predictors of reproductive success. Future research should aim to validate these findings under commercial, in vivo conditions.

## 5 Conclusion

Our results suggest that motility parameters alone after standard quality metrics are met are not accurate predictors of a boar's fertility potential for IVF. While motility and morphology assessments are important prerequisites for fertilization, they do not possess significant predictive power regarding fertility outcomes *in vitro*. Incorporating capacitation‐related biomarkers into semen evaluation protocols can enhance fertility predictions, shifting conventional semen analysis methods toward a more accurate representation of a boar's fertilizing potential. Currently, protocols for assessing sperm biomarker status require additional cost, time, and labor—factors that commercial swine operations may not feasibly accommodate on a daily basis. Therefore, methods for incorporating and simplifying this complex analysis, such as machine learning and label‐free approaches, should be further explored to make advanced semen evaluation more accessible and practical for widespread use. Importantly, sperm zinc signatures are conserved between boar and human, supporting the potential translation of these biomarkers to human assisted reproduction. Such conserved capacitation‐related indicators could complement current clinical semen assessments, improving the detection of subfertility in men who otherwise meet standard quality thresholds. Future work should focus on cross‐species validation to establish the robustness of zinc signatures and related biomarkers as universal indicators of fertility potential.

## Author Contributions

**Isabel Rodriguez:** methodology, software, validation, formal analysis, investigation, data curation, writing – original draft, writing – review and editing, funding acquisition, visualization. **Alexandra Keller:** writing – review and editing, investigation. **Lindsey Jennett:** writing – review and editing, investigation. **Megan Johnson:** writing – review and editing, investigation. **Ian Shofner:** writing – review and editing, investigation. **Mubashrah Mahmood:** writing – review and editing, investigation. **Bethany Redel:** conceptualization, methodology, supervision, writing – review and editing, resources. **Karl Kerns:** conceptualization, methodology, investigation, funding acquisition, writing – original draft, writing – review and editing, project administration, supervision, resources.

## Ethics Statement

Ethical review and approval were not required for this study. Boar sperm samples were obtained in collaboration with industry partners as surplus from routine semen collection for commercial breeding and were not collected specifically for research purposes. Porcine oocytes were recovered from ovaries collected at a local harvest facility as byproducts of standard food production; no animals were slaughtered for the purpose of this study. All sample handling followed established industry and laboratory protocols consistent with ethical and humane treatment of animals. In accordance with institutional and national guidelines, this work was exempt from Iowa State University Institutional Animal Care and Use Committee (IACUC) oversight.

## Consent

Patient consent statement is not applicable. This study did not involve human participants.

## Conflicts of Interest

The authors declare no conflicts of interest.

## Supporting information

Supplemental_materials_Clean.

## Data Availability

The data sets generated during the current study are available from the corresponding author on reasonable request. No centralized public repository currently supports the deposition of raw image‐based flow cytometry data sets.

## References

[mrd70085-bib-0001] Althouse, G. C. 2015. *Breeding Management in Pigs*. https://ucanr.edu/sites/default/files/2020-12/341992.pdf.

[mrd70085-bib-0002] Austin, C. 1951. “Observations on the Penetration of the Sperm Into the Mammalian Egg.” Australian Journal of Biological Sciences 4, no. 4: 581–596. 10.1071/BI9510581. 14895481

[mrd70085-bib-0003] Bae, J.‐W. , J.‐M. Hwang , W.‐J. Lee , et al. 2024. “Application of Sperm Motion Kinematics and Motility‐Related Proteins for Prediction of Male Fertility.” Theriogenology 218: 223–230. 10.1016/j.theriogenology.2024.02.007. 38359560

[mrd70085-bib-0004] Cassuto, N. G. , L. Ruoso , L. Prat‐Ellenberg , et al. 2024. “P‐023 Gene Expression in Human Sperm as Biomarkers and Tools for the Assessment of Male Infertility.” Supplement, Human Reproduction 39, no. Suppl_1: deae108.402. 10.1093/humrep/deae108.402.

[mrd70085-bib-0005] Chang, M. C. 1951. “Fertilizing Capacity of Spermatozoa Deposited Into the Fallopian Tubes.” Nature 168, no. 4277: 697–698. 10.1038/168697b0. 14882325

[mrd70085-bib-0006] Chang, Y. , D. Dai , Y. Li , et al. 2016. “Differences in the Expression of microRNAs and Their Predicted Gene Targets Between Cauda Epididymal and Ejaculated Boar Sperm.” Theriogenology 86, no. 9: 2162–2171. 10.1016/j.theriogenology.2016.07.012. 27527406

[mrd70085-bib-0007] Cheng, F. P. , A. Fazeli , W. F. Voorhout , A. Marks , M. M. Bevers , and B. Colenbrander . 1996. “Use of Peanut Agglutinin to Assess the Acrosomal Status and the Zona Pellucida‐Induced Acrosome Reaction in Stallion Spermatozoa.” Journal of Andrology 17, no. 6: 674–682. 10.1002/j.1939-4640.1996.tb01852.x. 9016398

[mrd70085-bib-0008] Conflitti, A. C. , G. Cicolani , A. Buonacquisto , et al. 2023. “Sperm DNA Fragmentation and Sperm‐Borne miRNAs: Molecular Biomarkers of Embryo Development?” International Journal of Molecular Sciences 24, no. 2: 1007. Article 2. 10.3390/ijms24021007. 36674527 PMC9864861

[mrd70085-bib-0009] Crowley, L. C. , A. P. Scott , B. J. Marfell , J. A. Boughaba , G. Chojnowski , and N. J. Waterhouse . 2016. “Measuring Cell Death by Propidium Iodide Uptake and Flow Cytometry.” Cold Spring Harbor Protocols 2016, no. 7: pdb.prot087163. 10.1101/pdb.prot087163. 27371595

[mrd70085-bib-0010] Escoffier, J. , F. Navarrete , D. Haddad , C. M. Santi , A. Darszon , and P. E. Visconti . 2015. “Flow Cytometry Analysis Reveals That Only a Subpopulation of Mouse Sperm Undergoes Hyperpolarization During Capacitation.” Biology of Reproduction 92, no. 5: 121. 10.1095/biolreprod.114.127266. 25855261 PMC4645980

[mrd70085-bib-0011] Ickowicz, D. , M. Finkelstein , and H. Breitbart . 2012. “Mechanism of Sperm Capacitation and the Acrosome Reaction: Role of Protein Kinases.” Asian Journal of Andrology 14, no. 6: 816–821. 10.1038/aja.2012.81. 23001443 PMC3720105

[mrd70085-bib-0012] Jin, S.‐K. , and W.‐X. Yang . 2016. “Factors and Pathways Involved in Capacitation: How Are They Regulated?.” Oncotarget 8, no. 2: 3600–3627. 10.18632/oncotarget.12274. PMC5356907 27690295

[mrd70085-bib-0013] Jodar, M. , C. Attardo‐Parrinello , A. Soler‐Ventura , et al. 2020. “Sperm Proteomic Changes Associated With Early Embryo Quality After ICSI.” Reproductive BioMedicine Online 40, no. 5: 700–710. 10.1016/j.rbmo.2020.01.004. 32444165

[mrd70085-bib-0015] Junahar, D. , R. Dwiputri , W. Adikusuma , et al. 2024. “Potential Biomarker Signatures in Male Infertility: Integrative Genomic Analysis.” Egyptian Journal of Medical Human Genetics 25, no. 1: 39. 10.1186/s43042-024-00512-7.

[mrd70085-bib-0016] Kerns, K. , M. Zigo , E. Z. Drobnis , M. Sutovsky , and P. Sutovsky . 2018. “Zinc Ion Flux During Mammalian Sperm Capacitation.” Nature Communications 9, no. 1: 2061. 10.1038/s41467-018-04523-y. PMC5970269 29802294

[mrd70085-bib-0017] Kerns, K. , M. Zigo , and P. Sutovsky . 2018. “Zinc: A Necessary Ion for Mammalian Sperm Fertilization Competency.” International Journal of Molecular Sciences 19, no. 12: 4097. 10.3390/ijms19124097. 30567310 PMC6321397

[mrd70085-bib-0018] Kwon, W.‐S. , M. S. Rahman , J.‐S. Lee , S.‐J. Yoon , Y.‐J. Park , and M.‐G. Pang . 2015. “Discovery of Predictive Biomarkers for Litter Size in Boar Spermatozoa*.” Molecular & Cellular Proteomics 14, no. 5: 1230–1240. 10.1074/mcp.M114.045369. 25693803 PMC4424395

[mrd70085-bib-0019] Ling, X. , P. Zou , L. Ao , et al. 2022. “Flow Cytometric Analysis of Biomarkers for Detecting Human Sperm Functional Defects.” Journal of Visualized Experiments 182: e63790. 10.3791/63790. 35532241

[mrd70085-bib-0020] Mateo‐Otero, Y. , F. Madrid‐Gambin , M. Llavanera , et al. 2023. “Sperm Physiology and In Vitro Fertilising Ability Rely on Basal Metabolic Activity: Insights From the Pig Model.” Communications Biology 6, no. 1: 344. 10.1038/s42003-023-04715-3. 36997604 PMC10063579

[mrd70085-bib-0021] Mehta, P. , and R. Singh . 2024. “Small RNAs: An Ideal Choice as Sperm Quality Biomarkers.” Frontiers in Reproductive Health 6: 1329760. 10.3389/frph.2024.1329760. 38406667 PMC10884189

[mrd70085-bib-0022] Mellagi, A. P. G. , K. J. Will , M. Quirino , I. C. Bustamante‐Filho , R. R. Ulguim , and F. P. Bortolozzo . 2023. “Update on Artificial Insemination: Semen, Techniques, and Sow Fertility.” Molecular Reproduction and Development 90, no. 7: 601–611. 10.1002/mrd.23643. 36063484

[mrd70085-bib-0023] Miller, R. , M. VerMilyea , L. Lipshultz , A. Olson , J. G. Kassab , and K. Brogaard . 2023. “Prospective Analysis of an Epigenetic Biomarker Predictive of Sperm Quality (SPERMQT).” Fertility and Sterility 120, no. 4: e328. 10.1016/j.fertnstert.2023.08.941.

[mrd70085-bib-0024] Ostermeier, G. C. , C. Cardona , M. A. Moody , et al. 2018. “Timing of Sperm Capacitation Varies Reproducibly Among Men.” Molecular Reproduction and Development 85, no. 5: 387–396. 10.1002/mrd.22972. 29521463 PMC6001750

[mrd70085-bib-0025] Pang, W.‐K. , S. Amjad , D.‐Y. Ryu , et al. 2022. “Establishment of a Male Fertility Prediction Model With Sperm RNA Markers in Pigs as a Translational Animal Model.” Journal of Animal Science and Biotechnology 13, no. 1: 84. 10.1186/s40104-022-00729-9. 35794675 PMC9261079

[mrd70085-bib-0026] Parvin, A. , G. Erabi , A. Alemi , et al. 2024. “Seminal Plasma Proteomics as Putative Biomarkers for Male Infertility Diagnosis.” Clinica Chimica Acta 561: 119757. 10.1016/j.cca.2024.119757. 38857670

[mrd70085-bib-0027] Petrocelli, H. , C. Batista , and J. Gosálvez . 2015. “Seasonal Variation in Sperm Characteristics of Boars in Southern Uruguay.” Revista Brasileira de Zootecnia 44: 1–7. 10.1590/S1806-92902015000100001.

[mrd70085-bib-0028] Petters, R. M. , and K. D. Wells . 1993. “Culture of Pig Embryos.” In Proceedings of the International Conference on Pig Reproduction, Bioscientifica Proceedings, Vol.14, 55–61. 10.1530/biosciprocs.14.005. 8145215

[mrd70085-bib-0029] Popwell, J. M. , and W. L. Flowers . 2004. “Variability in Relationships Between Semen Quality and Estimates of In Vivo and In Vitro Fertility in Boars.” Animal Reproduction Science 81, no. 1: 97–113. 10.1016/j.anireprosci.2003.08.007. 14749052

[mrd70085-bib-0041] R Core Team . 2024. R: A Language and Environment for Statistical Computing. R Foundation for Statistical Computing. https://www.R-project.org/.

[mrd70085-bib-0030] Redel, B. K. , L. D. Spate , and R. S. Prather . 2019. “In Vitro Maturation, Fertilization, and Culture of Pig Oocytes and Embryos.” In Comparative Embryo Culture, edited by J. R. Herrick , 2006, 93–103. Springer New York. 10.1007/978-1-4939-9566-0_6. 31230274

[mrd70085-bib-0031] Rodriguez‐Martinez, H. , J. Roca , M. Alvarez‐Rodriguez , and C. A. Martinez‐Serrano . 2022. “How Does the Boar Epididymis Regulate the Emission of Fertile Spermatozoa?.” Animal Reproduction Science 246: 106829. 10.1016/j.anireprosci.2021.106829. 34452796

[mrd70085-bib-0032] Sáez‐Espinosa, P. , N. Huerta‐Retamal , L. Robles‐Gómez , et al. 2020. “Influence of In Vitro Capacitation Time on Structural and Functional Human Sperm Parameters.” Asian Journal of Andrology 22, no. 5: 447. 10.4103/aja.aja_104_19. 31621655 PMC7523607

[mrd70085-bib-0034] Shike, J. 2020. “Debunking Traditional Semen Quality Standards.” *Pork Business*. https://www.porkbusiness.com/news/hog-production/debunking-traditional-semen-quality-standards.

[mrd70085-bib-0035] Sutovsky, P. , M. Aarabi , A. Miranda‐Vizuete , and R. Oko . 2015. “Negative Biomarker‐Based Male Fertility Evaluation: Sperm Phenotypes Associated With Molecular‐Level Anomalies.” Asian Journal of Andrology 17, no. 4: 554. 10.4103/1008-682X.153847. 25999356 PMC4492044

[mrd70085-bib-0036] Tollner, T. L. , C. A. VandeVoort , A. I. Yudin , C. A. Treece , J. W. Overstreet , and G. N. Cherr . 2009. “Release of DEFB126 From Macaque Sperm and Completion of Capacitation Are Triggered by Conditions That Simulate Periovulatory Oviductal Fluid.” Molecular Reproduction and Development 76, no. 5: 431–443. 10.1002/mrd.20964. 18937315

[mrd70085-bib-0037] USDA ERS . n.d. “Sector at a Glance.” Accessed July 26, 2024. https://www.ers.usda.gov/topics/animal-products/hogs-pork/sector-at-a-glance/.

[mrd70085-bib-0038] Westerman, R. 2020. “Biomarkers for Demographic Research: Sperm Counts and Other Male Infertility Biomarkers.” Biodemography and Social Biology 65, no. 1: 73–87. 10.1080/19485565.2019.1706150. 32065536

[mrd70085-bib-0039] Zigo, M. , K. Kerns , S. Sen , et al. 2022. “Zinc Is a Master‐Regulator of Sperm Function Associated With Binding, Motility, and Metabolic Modulation During Porcine Sperm Capacitation.” Communications Biology 5, no. 1: 538. 10.1038/s42003-022-03485-8. 35660793 PMC9166710

[mrd70085-bib-0040] Zigo, M. , P. Maňásková‐Postlerová , D. Zuidema , et al. 2020. “Porcine Model for the Study of Sperm Capacitation, Fertilization and Male Fertility.” Cell and Tissue Research 380, no. 2: 237–262. 10.1007/s00441-020-03181-1. 32140927

