# Supplementary material for: Capacitation‐Induced Zinc Ion Flux and Sperm Plasma Membrane Remodeling Predict Porcine In Vitro Fertilization Cleavage Success
Source: Mol Reprod Dev. 2026 Jan 21;93(1):e70085. doi: 10.1002/mrd.70085 (PMC12820604; doi:10.1002/mrd.70085)
Supplement: Supplementary file 1 — Supplemental_materials_Clean. [file MRD-93-e70085-s001.docx]

**Supplemental Materials**

| **Variable** | **Statistical test** | **Correlation coefficient** | ***p*** |
| --- | --- | --- | --- |
| BCF 0 hr | Pearson | -0.416440532 | 0.000934279 |
| BCF Δ1-0 hr | Pearson | 0.471827971 | 0.000141407 |
| BCF Δ4-0 hr | Pearson | 0.38116907 | 0.002658085 |
| BCF Δ4-1 hr | Spearman | -0.267668068 | 0.038675196 |
| DAP 0 hr | Pearson | -0.348822538 | 0.006304669 |
| DAP Δ1-0 hr | Pearson | 0.312294299 | 0.01513094 |
| DAP Δ4-0 hr | Pearson | 0.338499347 | 0.008158461 |
| DCL 0 hr | Pearson | -0.266889913 | 0.039266152 |
| DCL Δ1-0 hr | Pearson | 0.34261145 | 0.007369727 |
| DCL Δ4-1 hr | Spearman | -0.318284773 | 0.013197918 |
| Distal Droplet 1 hr | Spearman | 0.362874655 | 0.004378641 |
| Distal Droplet Δ4-1 hr | Pearson | -0.349659608 | 0.006171984 |
| DSL 0 hr | Pearson | -0.342632419 | 0.007365882 |
| DSL Δ1-0 hr | Pearson | 0.264669596 | 0.0409935 |
| DSL Δ4-0 hr | Pearson | 0.345698869 | 0.00682213 |
| HAC 0 hr | Pearson | -0.257499205 | 0.047004982 |
| HAC Δ1-0 hr | Pearson | 0.384732677 | 0.00240379 |
| HAC Δ4-0 hr | Pearson | 0.310597389 | 0.015720779 |
| Immotile 0 hr | Spearman | 0.265423525 | 0.040400066 |
| Immotile Δ1-0 hr | Pearson | -0.39466864 | 0.001805364 |
| LIN 0 hr | Pearson | -0.261529796 | 0.043542764 |
| LIN Δ4-0 hr | Pearson | 0.275396983 | 0.033195327 |
| Local Motility 1 hr | Spearman | 0.318441423 | 0.013150365 |
| PI- 0 hr | Spearman | -0.58450979 | 9.41401E-07 |
| PI- 1 hr | Spearman | 0.328257008 | 0.010450326 |
| PI- 4 hr | Spearman | 0.482575596 | 9.43073E-05 |
| PI- Δ1-0 hr | Spearman | 0.579062925 | 1.2536E-06 |
| PI- Δ4-0 hr | Spearman | 0.57367164 | 1.65606E-06 |
| PI- Δ4-1 hr | Spearman | 0.422326595 | 0.000775881 |
| PNA+ 0 hr | Spearman | 0.370692535 | 0.003549833 |
| PNA+ 4 hr | Spearman | 0.272120946 | 0.035433408 |
| Progressive Circular Motility Δ4-0 hr | Spearman | 0.439466371 | 0.000442996 |
| Progressive Circular Motility 0 hr | Spearman | -0.375845923 | 0.003082588 |
| Progressive Circular Motility Δ4-1 hr | Spearman | 0.310414457 | 0.015785526 |
| Progressive Circular Motility 4 hr | Spearman | 0.299026773 | 0.020291882 |
| Progressive Composite Score 0 hr | Pearson | -0.255299817 | 0.048987361 |
| Progressive Composite Score Δ1-0 hr | Pearson | 0.352887618 | 0.005682957 |
| Progressive Motility 0 hr | Spearman | -0.283042467 | 0.028427052 |
| Progressive Motility Δ1-0 hr | Pearson | 0.361035316 | 0.004596846 |
| Proximal Droplet 0 hr | Spearman | 0.295413179 | 0.02193288 |
| Rapid Motility Δ1-0 hr | Pearson | 0.274373835 | 0.033881294 |
| Slow Motility 1 hr | Spearman | 0.257117849 | 0.047343922 |
| Slow Motility Δ4-1 hr | Spearman | -0.304552027 | 0.017984894 |
| Total Composite Score Δ1-0 hr | Pearson | 0.381276006 | 0.002650119 |
| Total Motility 0 hr | Spearman | -0.265423525 | 0.040400066 |
| Total Motility Δ1-0 hr | Pearson | 0.39466864 | 0.001805364 |
| VAP 0 hr | Pearson | -0.265330178 | 0.040473155 |
| VAP Δ1-0 hr | Pearson | 0.26008266 | 0.044760867 |
| VAP Δ4-0 hr | Pearson | 0.264218351 | 0.041352112 |
| VCL Δ1-0 hr | Pearson | 0.277027631 | 0.032126007 |
| VSL 0 hr | Pearson | -0.265036692 | 0.040703659 |
| VSL Δ4-0 hr | Pearson | 0.275154421 | 0.033356897 |
| WOB 0 hr | Pearson | -0.379270499 | 0.002803113 |
| WOB Δ4-0 hr | Pearson | 0.314980035 | 0.014236189 |
| ZnSig1 4 hr | Spearman | -0.365884843 | 0.004041216 |
| ZnSig2 1 hr | Spearman | 0.351239445 | 0.00592822 |
| ZnSig2 4 hr | Spearman | 0.269147403 | 0.037572032 |
| ZnSig2 Δ4-0 hr | Spearman | 0.371276128 | 0.003493944 |
| ZnSig2 Δ4-1 hr | Spearman | -0.340095603 | 0.007843956 |
| ZnSig3 1 hr | Spearman | -0.362633398 | 0.004406731 |
| ZnSig3 4 hr | Spearman | -0.284515344 | 0.027577866 |
| ZnSig3 Δ1-0 hr | Spearman | -0.441418414 | 0.00041482 |
| ZnSig3 Δ4-0 hr | Spearman | -0.374610943 | 0.003189284 |
| ZnSig3 Δ4-1 hr | Spearman | 0.323616056 | 0.011660312 |
| ZnSig4 0 hr | Spearman | -0.276122725 | 0.032715805 |
| ZnSig4 Δ1-0 hr | Spearman | 0.25783682 | 0.046706581 |

**Supplemental Table 1. Correlation coefficients and their respective *p-value*s** **.** This table corresponds to **Figure 2.**

| Fertility groups t-test results | |
| --- | --- |
| **Variable** | ***p*** |
| PNA+ 0 hr | 0.02898 |
| PNA+ 4 hr | 0.013648 |
| PI- 0 hr | 8.6 x 10^-7^ |
| PI- 4 hr | 0.000831 |
| ZnSig1 4 hr | 0.007416 |
| ZnSig3 Δ1-0 hr | 0.043283 |

**Supplemental Table 2. High vs low fertility group t-test results.** This table corresponds with **Figure 3.**

| **Category** | **Variables** |
| --- | --- |
| Motility | Total Motility (%), Progressive Motility (%), Rapid Motility (%), Slow Motility (%), Progressive Circular Motility (%), Immotile (%), Local Motility (%). |
| Kinematics | Total Composite Score (%), Progressive Composite Score (%), DCL, DSL, DAP, VCL, VSL, VAP, BCF, ALH, HAC, STR, LIN, WOB. |
| Morphology | Bent Tail (%), Distal Droplet (%), Proximal Droplet (%), Total Defects (%). |
| Biomarkers | PNA (%), PI (%), Zn signatures 1-4 (%) |

**Supplemental Table 3. Variables included in PCA-based LM and LMER models.** All variables listed above were included in the PCA to generate category-specific PC1 scores for linear models (LM) and mixed-effects models (LMER). For the general LM and LMER analyses, each variable was included across all measured timepoints (0 hr, 1 hr, 4 hr, and the three corresponding deltas previously described). For timepoint-specific LM and LMER analyses, only variables measured at the respective timepoint were incorporated. This table corresponds with (**Figures 4-6**)

| **Model** | **Timepoint** | **LM (R^2^)** | **LM (*p)*** | **LMER (Marginal R^2^)** |
| --- | --- | --- | --- | --- |
| Combined | All | 0.46869795 | 3.89 x 10^-7^ | 0.290417731 |
|  | 0 hr | 0.28325349 |  | 0.110021842 |
|  | 1 hr | 0.13478888 |  | 0.053643758 |
|  | 4 hr | 0.16087517 |  | 0.077082124 |
|  | Delta (Δ) | 0.49216577 |  | 0.362486465 |
| Biomarkers | All | 0.43021634 | 1.3 x 10-8 | 0.288384267 |
|  | 0 hr | 0.22121183 |  | 0.05662711 |
|  | 1 hr | 0.01594169 |  | 0.00023698 |
|  | 4 hr | 0.1304406 |  | 0.049554205 |
|  | Delta (Δ) | 0.42504826 |  | 0.259090339 |
| Motility | All | 0.04818315 | 0.092 | 0.018252176 |
|  | 0 hr | 0.07292121 |  | 0.005913606 |
|  | 1 hr | 0.02657062 |  | 0.024577406 |
|  | 4 hr | 3.3428E-05 |  | 1.00807E-05 |
|  | Delta (Δ) | 0.10227988 |  | 0.020207664 |
| Kinematics | All | 0.10384533 | 0.012 | 0.000617849 |
|  | 0 hr | 0.09606697 |  | 0.012323183 |
|  | 1 hr | 0.0033627 |  | 0.012681805 |
|  | 4 hr | 0.00011657 |  | 2.11353E-05 |
|  | Delta (Δ) | 0.1092399 |  | 0.023757322 |
| Morphology | All | 0.02456003 | 0.232 | 0.002588205 |
|  | 0 hr | 0.03707707 |  | 0.004374257 |
|  | 1 hr | 0.0566294 |  | 0.006969776 |
|  | 4 hr | 0.01462367 |  | 0.005069337 |
|  | Delta (Δ) | 0.00194154 |  | 0.002711633 |

**Supplemental Table 4. Linear model and mixed-effects model performance across timepoints for each parameter category.** This table corresponds with **Figures 4-6**.


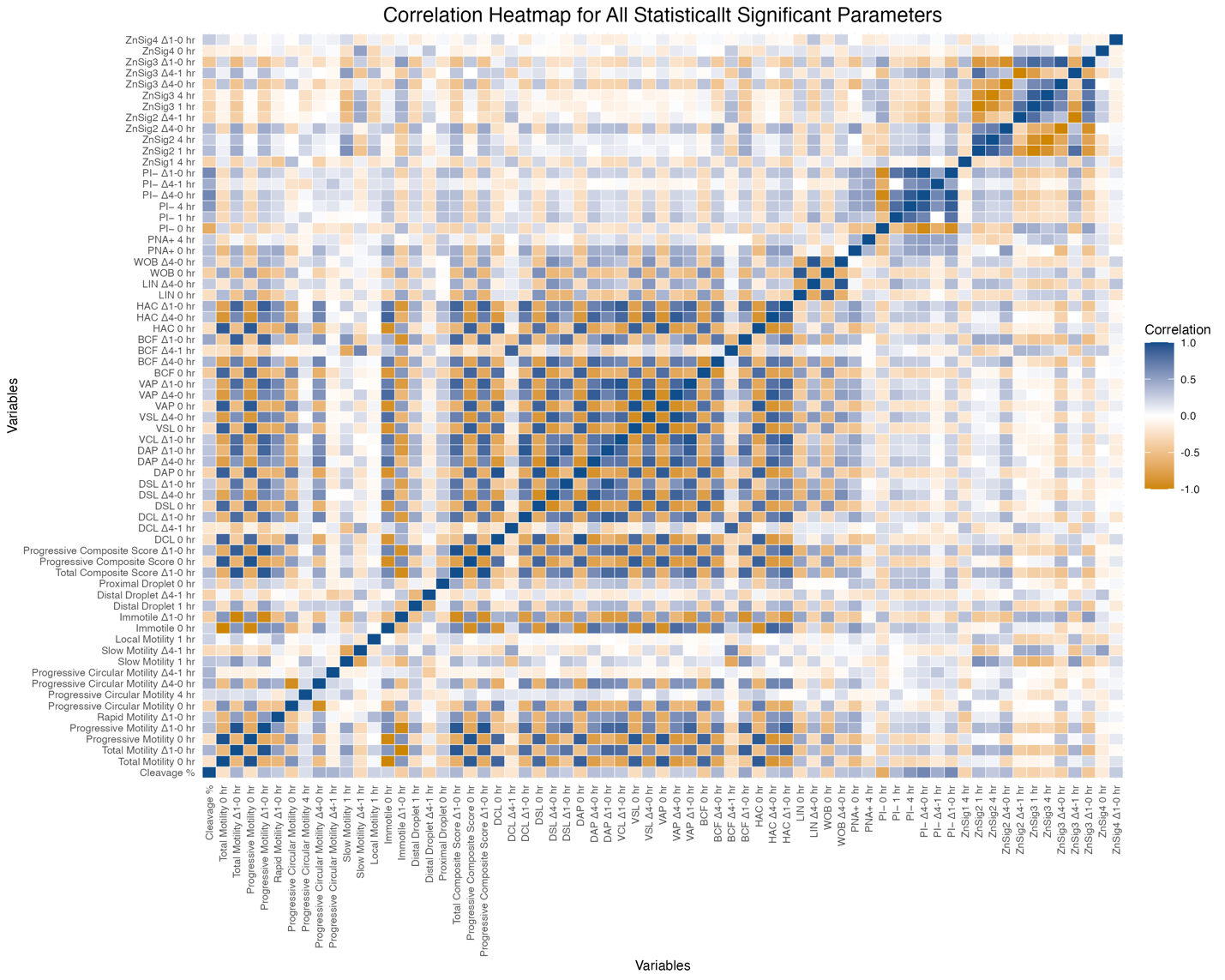


**Supplemental Figure 1. Correlation Heatmap for all statistically significant parameters correlated with cleavage rates.** Pearson or Spearman correlation coefficients were calculated based on the data distribution between cleavage percentage and all sperm parameters, as well as their delta values, at the specified time points (0 hr, 1 hr post-IVC, and 4 hr post-IVC). Sixty-six parameters were found statistically significant. Blue indicates positive correlations with cleavage percentage, while orange indicates negative correlations with cleavage percentage. Specific correlation test used for each parameter, along with specific correlation coefficients and *p-values* are in **Supplemental Table 1**.


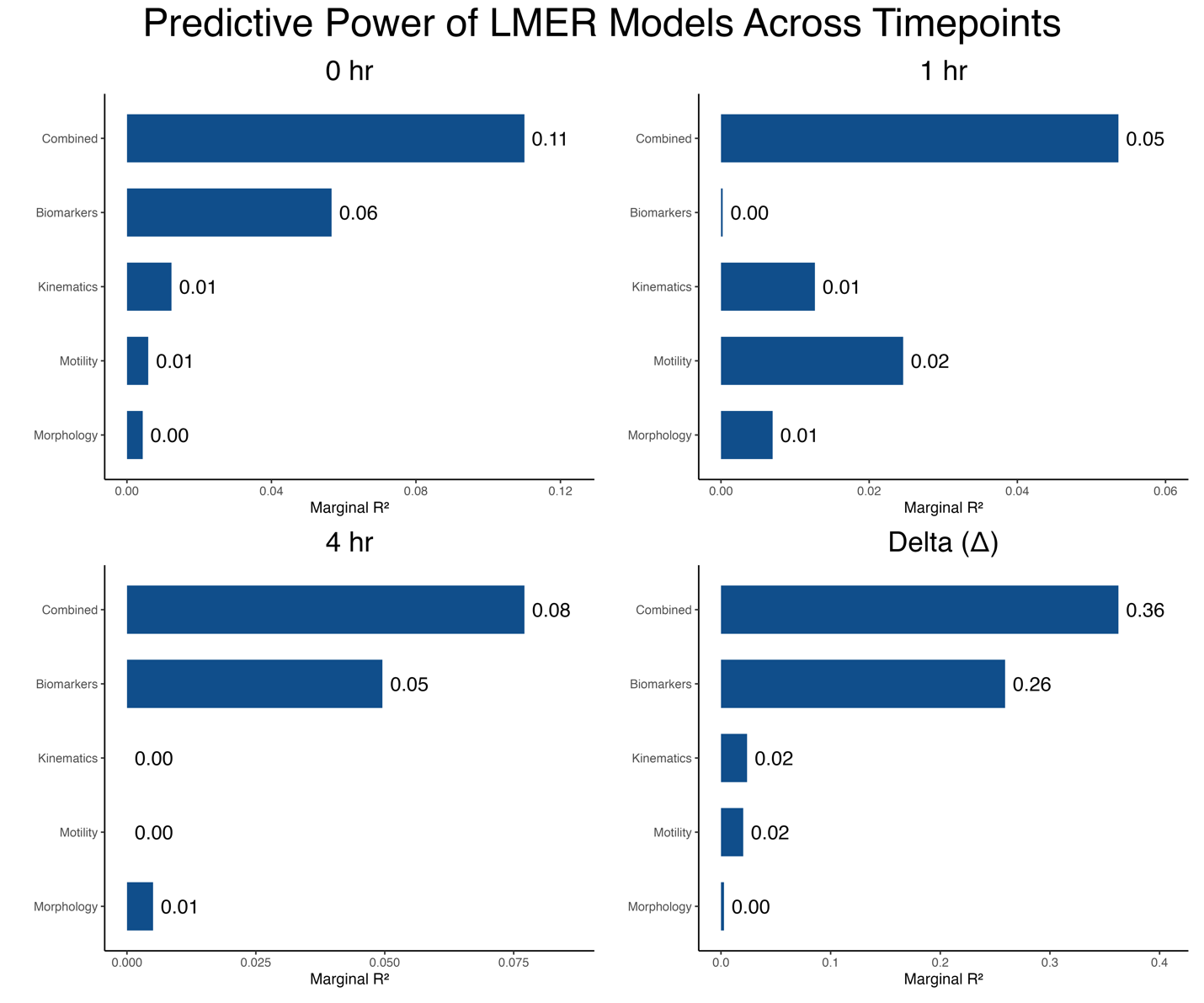


**Supplemental Figure 2. Predictive power of linear mixed-effect models across timepoints.** Variance explained (marginal R^2^) by LMER models for five parameter categories (motility, kinematics, morphology, biomarkers, and the combined model) evaluated at 0 hr, 1 hr post-IVC, 4 hr post-IVC, and for delta values representing dynamic changes between timepoints. Delta models displayed the strongest R^2^ values, indicating that dynamic changes are more predictive than static parameters.

**
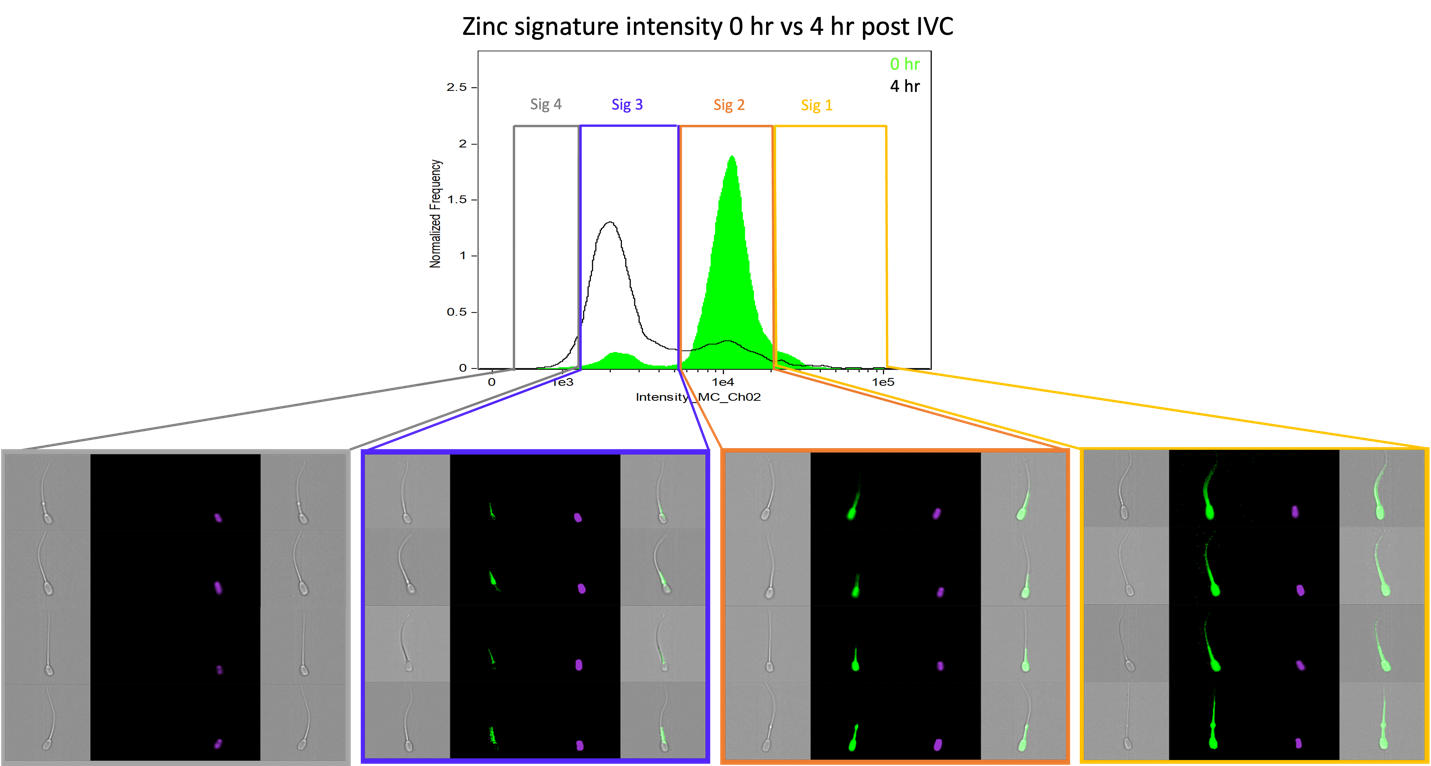
**

**Supplemental Figure 3. Image-Based Flow Cytometry Zinc Signatures.** Boar sperm zinc signatures 1 through 4 gated by FZ3 intensity for 0 hr (filled green) and 4 hr post IVC (black line).


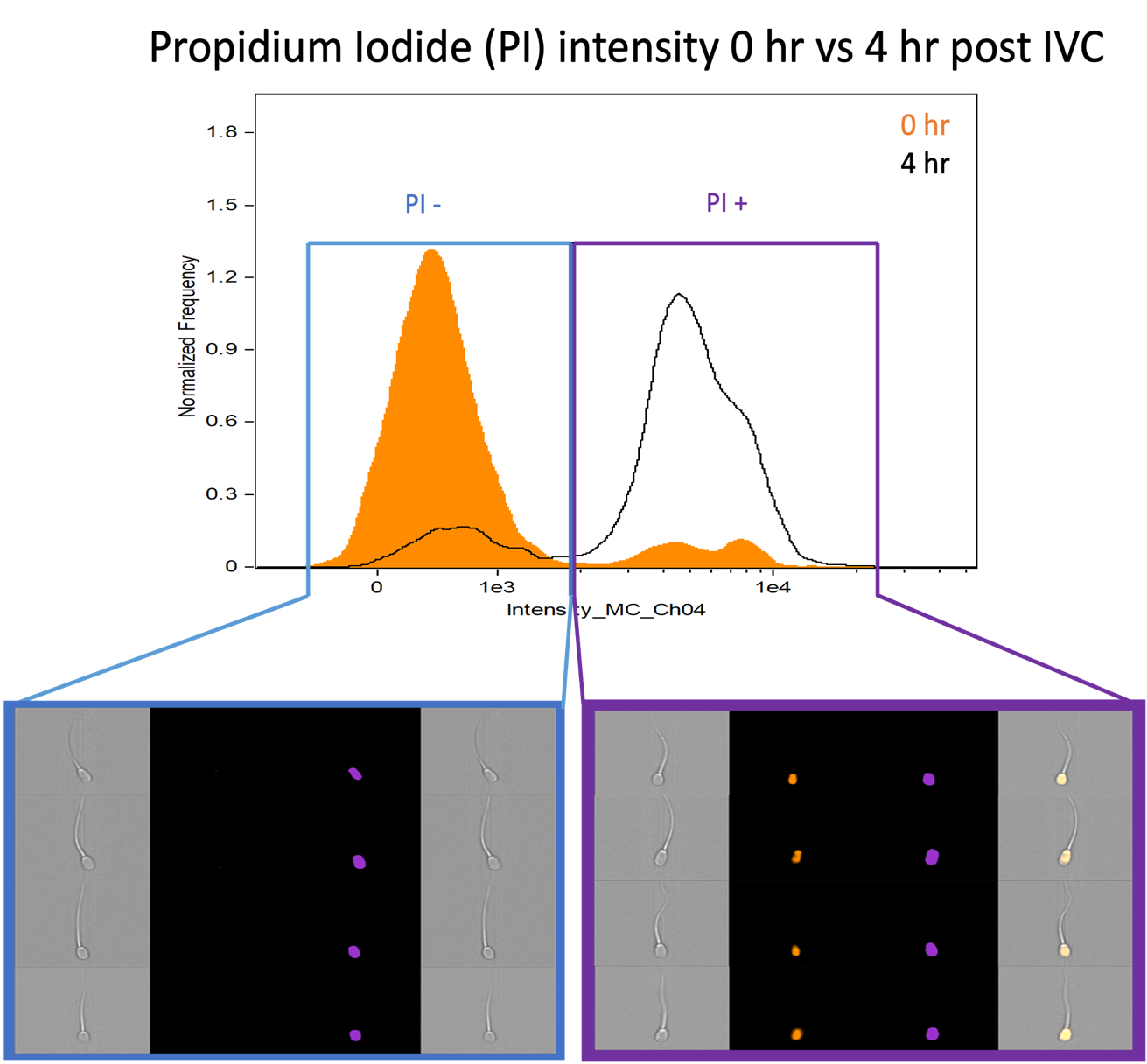


**Supplemental Figure 4. Image-Based Flow Cytometry Plasma Membrane Integrity.** Plasma membrane integrity for boar sperm cells gated by propidium iodide (PI) intensity for 0 hr (filled orange), and 4 hr post IVC (black line).


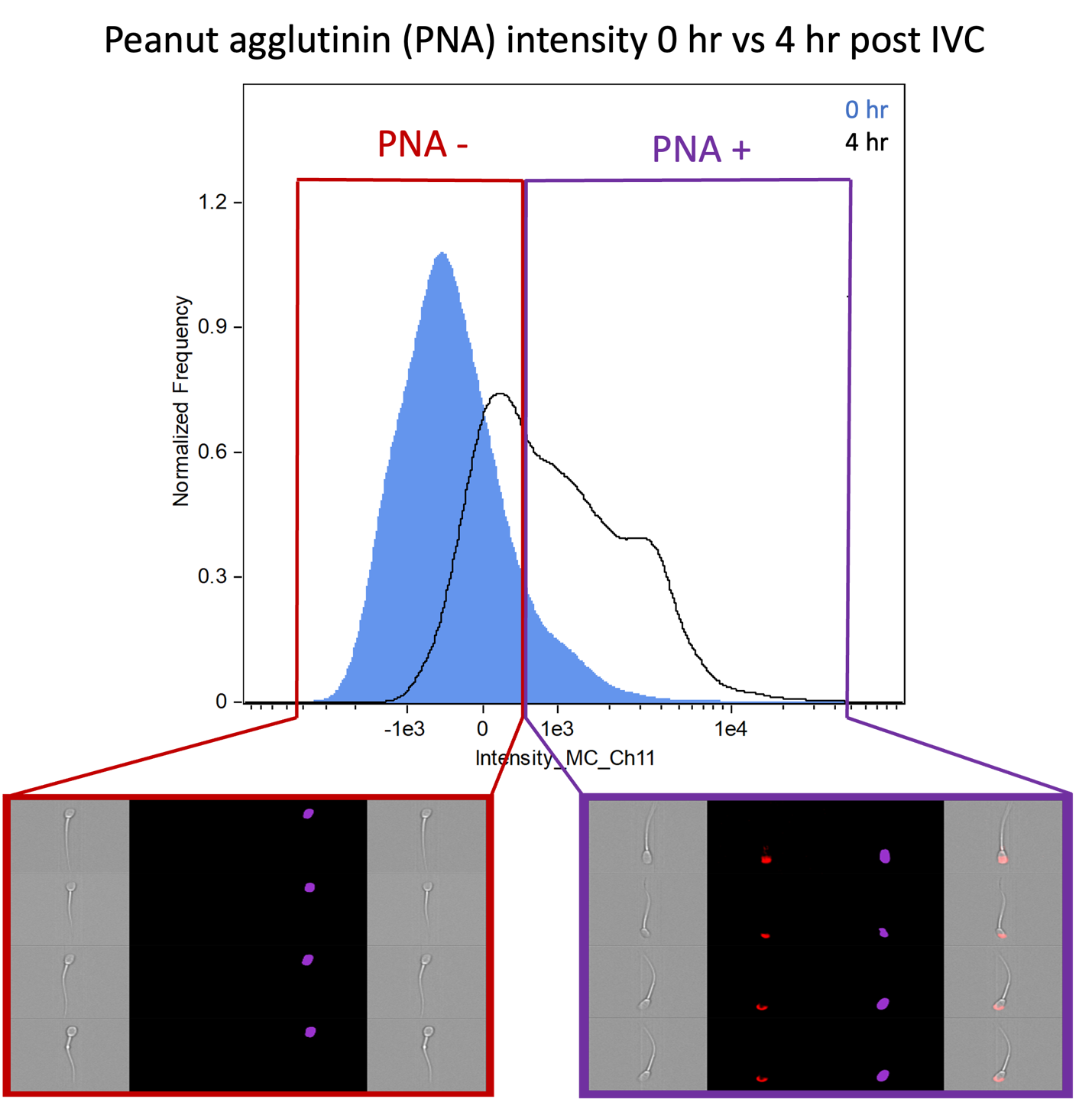


**Supplemental Figure 5. Image-Based Flow Cytometry Acrosome Status.** Boar sperm acrosome status gated by Peanut agglutinin (PNA) intensity for 0 hr (filled blue), and 4 hr post IVC (black line).
